# *Caenorhabditis elegans* nicotinic acetylcholine receptors are required for nociception

**DOI:** 10.1016/j.mcn.2014.02.001

**Published:** 2014-03

**Authors:** Emiliano Cohen, Marios Chatzigeorgiou, Steven J. Husson, Wagner Steuer-Costa, Alexander Gottschalk, William R. Schafer, Millet Treinin

**Affiliations:** a Department of Medical Neurobiology, Institute for Medical Research Israel–Canada, Hebrew University — Hadassah Medical School, Jerusalem 91120, Israel; b Cell Biology Division, MRC Laboratory of Molecular Biology, Hills Road, Cambridge UK; c Functional Genomics and Proteomics, KU Leuven, Naamsestraat 59, B-3000 Leuven, Belgium; d SPHERE — Systemic Physiological & Ecotoxicological Research, Department of Biology, University of Antwerp, Groenenborgerlaan 171/U7, B-2020 Antwerp, Belgium; e Buchmann Institute for Molecular Life Sciences and Institute of Biochemistry, Goethe-University Frankfurt, Max-von-Laue-Str. 15, D-60438 Frankfurt, Germany

**Keywords:** *Caenorhabditis elegans*, Polymodal nociceptors, Nicotinic acetylcholine receptors, Acetylcholine, Calcium

## Abstract

Polymodal nociceptors sense and integrate information on injurious mechanical, thermal, and chemical stimuli. Chemical signals either activate nociceptors or modulate their responses to other stimuli. One chemical known to activate or modulate responses of nociceptors is acetylcholine (ACh). Across evolution nociceptors express subunits of the nicotinic acetylcholine receptor (nAChR) family, a family of ACh-gated ion channels. The roles of ACh and nAChRs in nociceptor function are, however, poorly understood. *Caenorhabditis elegans* polymodal nociceptors, PVD, express nAChR subunits on their sensory arbor. Here we show that mutations reducing ACh synthesis and mutations in nAChR subunits lead to defects in PVD function and morphology. A likely cause for these defects is a reduction in cytosolic calcium measured in ACh and nAChR mutants. Indeed, overexpression of a calcium pump in PVD mimics defects in PVD function and morphology found in nAChR mutants. Our results demonstrate, for the first time, a central role for nAChRs and ACh in nociceptor function and suggest that calcium permeating via nAChRs facilitates activity of several signaling pathways within this neuron.

## Introduction

In animals, injurious signals are detected by specialized sensory neurons called nociceptors. These neurons are often polymodal sensing both high-threshold mechanical stimuli and noxious temperatures, most also respond to chemical stimuli. These chemicals include protons, ATP, bradykinin, prostaglandins, other cytokines, and neurotransmitters (Woolf and Ma, 2007). Responses of nociceptors to noxious stimuli and the ensuing behavioral and physiological responses show a great degree of plasticity; evident at the sensory level by altered response threshold or intensity. Indeed, sensitization following injury is a unique property of polymodal nociceptors and is markedly different from the rapidly desensitizing responses of other sensory neurons. This sensitization is likely a result of chemicals, present in the environment following injury or inflammation, that act via signaling pathways within nociceptors to enhance responses to concurrent or future noxious stimuli (Fischer et al., 2010, Gold and Gebhart, 2010). For example, serotonin and bradykinin whose extra-cellular levels increase following inflammation are known to cause sensitization via regulation of activity or activation thresholds of nociceptor-expressed ion-channels (Beck and Handwerker, 1974, Loyd et al., 2013, Petho and Reeh, 2012).

Early analysis of chemical excitants of nociceptors identified ACh as a pain-producing substance (Armstrong et al., 1953). Further analysis showed that nAChRs mediate some of the effects of ACh on nociceptors (Bernardini et al., 2001). Indeed, mammalian nociceptors express multiple nAChR subunits (Damaj and Flores, 2001, Genzen et al., 2001, Rau et al., 2004, Spies et al., 2006). Analysis of the role of nAChRs in nociception, however, is hindered by the many roles and wide expression of nAChRs. For example, sites of action of analgesic drugs targeting nAChRs include targets in the central nervous system, sensory endings of nociceptors, and non-neuronal cells mediating inflammatory-responses (Damaj and Flores, 2001, Vincler et al., 2006).

Responding appropriately to noxious and injurious signals is essential for survival, as evidenced by conservation of polymodal nociceptors in evolution. Polymodal nociceptors are found in both vertebrates and invertebrates and are conserved for function, molecular determinants and morphology (Hall and Treinin, 2011). Recently, the *Caenorhabditis elegans* PVD and FLP neurons emerged as well-conserved models for polymodal nociceptors. These two pairs of neurons, FLP in the head and PVD in the midbody, sense high-threshold mechanical stimuli and temperature; PVD sense noxious cold while FLP sense noxious heat (Chatzigeorgiou and Schafer, 2011, Chatzigeorgiou et al., 2010). Like mammalian nociceptors PVD and FLP express several nAChR subunits (Smith et al., 2010, Treinin et al., 1998). These subunits, however, are not conserved in evolution; as DEG-3 and DES-2, shown to express in PVD, belong to a *C. elegans* specific group of nAChR subunits (Jones and Sattelle, 2003, Treinin et al., 1998).

Conservation between PVD and mammalian nociceptors enables analysis of the role of ACh and nAChRs in nociceptor function. We examined mutants affecting DEG-3, DES-2, and ACh synthesis for defects in PVD function or morphology and demonstrated roles of nAChR subunits and ACh in PVD function and development. Furthermore, this work shows that ACh and ACh-gated ion-channels enhance the response amplitude of PVD to noxious stimuli. We suggest that functions of nAChRs in PVD represent an evolutionarily conserved mechanism for facilitating responses of polymodal nociceptors.

## Results

### DEG-3 and DES-2 localize to PVD sensory dendrites

The DEG-3 and DES-2 nAChR subunits are together required for formation of a functional ACh- and choline-gated ion-channel. *deg-3* and *des-2* are encoded by a single operon that was shown to express in several neurons including PVD and FLP neurons (Treinin et al., 1998). Neurotransmitter gated channels are presumed to function in synapses, however, anti-DEG-3 staining did not show synaptic localization, instead it demonstrated localization of DEG-3 to sensory dendrites and revealed the multi-dendritic nature of PVD and FLP (Halevi et al., 2002, Yassin et al., 2001). Thus, suggesting a role for DEG-3 in sensory transduction.

**Fig. 1:**
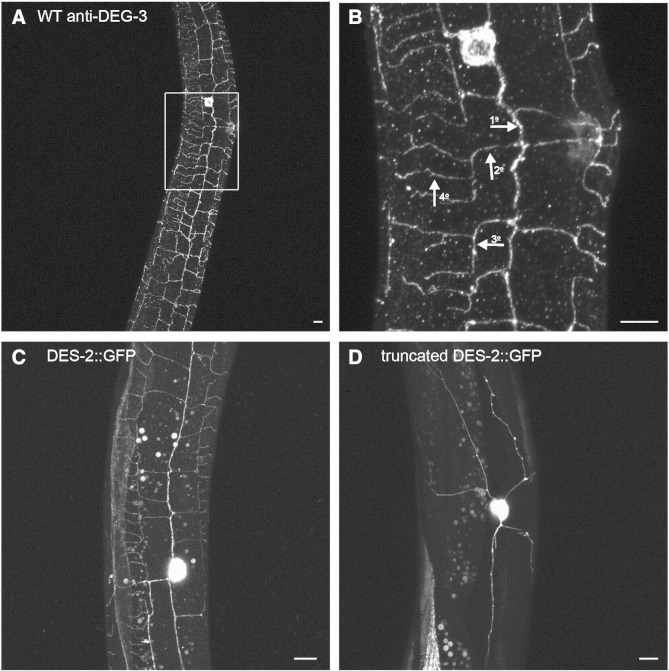
DEG-3 and DES-2 localization in PVD's sensory arbor. A) Anti-DEG-3 antibody staining. B) Magnification of boxed area in (A). Arrows and numbers indicate representative primary (1°), secondary (2°), tertiary (3°), and quaternary branches (4°). C) Full-length DES-2::GFP fusion localization in PVD. D) PVD localization of a truncated DES-2::GFP fusion. Shown are images of late L4-young adults, scale bar 5 μm.

PVD neurons are multi-dendritic neurons having a complex arborization pattern. Each of the two PVD neurons extends one axon to the ventral cord and two longitudinal primary dendrites, one extending to the tail and the other to the head. Secondary branches growing ventrally or dorsally from primary dendrites each form a menorah like structure whose terminal branches, quaternary branches, lie in between the body muscles and the hypodermis (Albeg et al., 2011, Oren-Suissa et al., 2010). Fig. 1A,B shows that DEG-3 decorates the entire dendritic arbor of PVD, primary, secondary, tertiary, and quaternary branches. Similarly, a full length DES-2::GFP fusion also localizes to the entire sensory arbor of PVD (Fig. 1C). Interestingly, a DES-2::GFP fusion lacking the C-terminus (129 amino acids starting at P421 within the large intracellular loop) does not localize to terminal branches (Fig. 1D). Absence of the truncated DES-2::GFP from PVD terminal branches (Fig. 1D) is not a result of transgene-induced defects in their development, as they can be visualized using anti-DEG-3 staining of transgenic animals (results not shown). Thus, terminal dendrite localization of DES-2 is not simply a result of lateral diffusion within PVD membranes. Instead, trafficking to terminal dendrites is likely to require assembly of full-length DES-2 with DEG-3. Indeed, assembly of DEG-3 with DES-2 is required for DEG-3 localization to PVD sensory arbor (Yassin et al., 2002). Therefore, localization of both DEG-3 and DES-2 to PVD terminal dendrites is likely to be a result of active trafficking of mature fully assembled receptors. Such active trafficking is consistent with a requirement for DEG-3 and DES-2 in PVD's sensory arbors.

### DEG-3 and DES-2 are required for the harsh-touch response

**Fig. 2:**
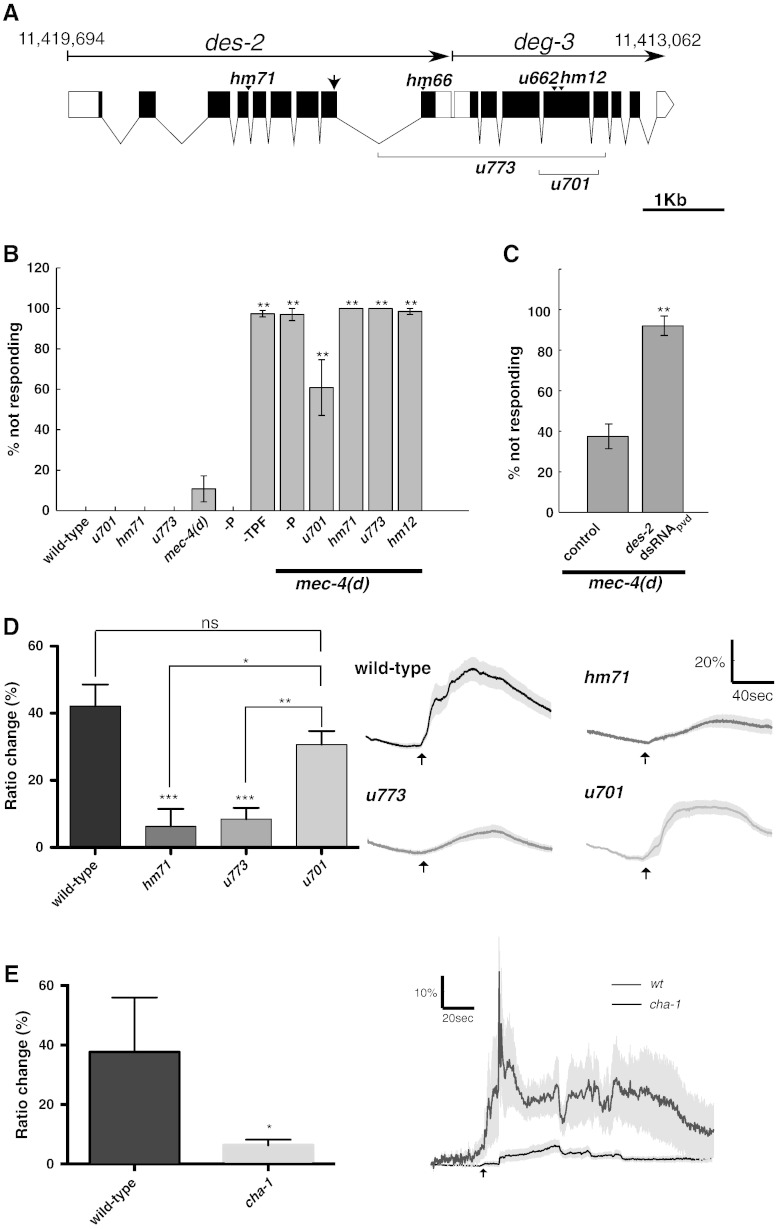
The harsh touch response depends on DES-2. A) A diagram of the DEG-3 and DES-2 expressing operon. Arrow heads indicate position of point mutations, lines indicate extent and position of deletions, and an arrow indicates the site at which DES-2 is truncated in the DES-2::GFP transgene. Numbers indicate genomic position of the first and last nucleotides of the operon. Scale bar indicates 1 Kb. B) Young adults were prodded with a platinum wire and the percent of animals not responding by accelerated movement or by reversals were recorded. Strains examined are wild-type (N2); mutants in *deg-3* and/or *des-2*; animals lacking TRNs (*mec-4(d)*), PVDs (-P), or TRNs, PVD, and FLP (-TPF); and animals combining the *mec-4(d)* mutation with: PVD elimination (-P) or different *deg-3* and/or *des-2* alleles (N = 3–10,10–20 animals in each experiment). Asterisks indicate significant difference relative to wild-type, *mec-4(d)* and -P animals. In addition, *mec-4(d);deg-3(u701)* is significantly different from all other strains (p < 0.01). C) Animals lacking TRNs (*mec-4 (d)*) and expressing empty vector or *des-2* dsRNA in PVD, were examined for the harsh touch response (N = 4–5, 8–12 animals in each experiment). Effects of expressing *des-2* dsRNA relative to animals expressing empty vector is significant using Wilcoxon analysis (p < 0.016). D). Left, averaged peak calcium response in wild-type, *des-2(hm71)*, *des-2deg-3(u773)*, and *deg-3(u701)*, N = 18, 12, 22, 22 respectively. Right, averaged traces of calcium responses with s.e.m. Arrows indicate time of harsh touch application. E) Left, averaged peak calcium response in wild-type and *cha-1(p1152)*, N = 5,13 respectively. Right, averaged traces of calcium responses with s.e.m. Arrows indicate time of harsh touch application. Significant differences relative to wild-type or as indicated are * — p < 0.05, ** — p < 0.01, *** — p < 0.001 one-way ANOVA.

*C. elegans* responds to mechanical stimuli by altering its locomotion. The response to low threshold mechanical stimulation to the animal's body (touching with a hair or tapping the plate) requires the six touch receptor neurons (TRNs) (Chalfie and Sulston, 1981, Wicks and Rankin, 1995). The response to high-threshold mechanical stimulation (prodding the mid-body with a wire pick) is not eliminated in animals lacking TRNs (*mec-4(d)* mutants) or PVD. Elimination of both TRNs and PVD, however, eliminates the response to such prodding (Albeg et al., 2011, Way and Chalfie, 1989). Redundancy between PVD and TRNs may explain why mutants in DEG-3 or DES-2 are not harsh touch defective (Fig. 2A,B and Table 1). Therefore, to examine the role of DEG-3 and DES-2 in the PVD-mediated behavioral response to harsh touch we combined mutations in these genes with the *mec-4(d)* mutation. Analysis of these double mutants showed that two mutations *u773*, a deletion affecting *deg-3* and *des-2*, and *hm71*, a *des-2* a point mutation leading to truncation of DES-2 (both likely to be DES-2 loss of function *(lf)* mutations) eliminate the behavioral response to harsh touch (Fig. 2A,B and Table 1). Moreover, analysis of *mec-4(d)* animals expressing *des-2* dsRNA in PVD shows significant reduction relative to control (*mec-4(d)* expressing empty vector) in the response to harsh touch (Fig. 2C). We note that high copy number of the *ser-2prom3* promoter is sufficient for some defects in PVD function (control in Fig. 2C). Nevertheless, the dsRNA experiments show that DES-2 expression in PVD is required for the PVD-mediated harsh touch response. A deletion mutation eliminating *deg-3* alone, *u701*, has a smaller, although significant, effect on the harsh-touch response relative to mutations eliminating *des-2* (Fig. 2A,B and Table 1). Therefore, while both DEG-3 and DES-2 are needed for the harsh touch response, DES-2 appears to have a more important role in this response.

**Table 1:** Mutations in nAChR subunits.

Gene(s)	Allele	Type of mutation ⁎	Position⁎⁎ (nucleotides)	Amino-acid change ⁎⁎⁎
*des-2*	*hm71*	Splice site mutation (*lf*)	V:11,417,868	Truncation after K210
*des-2*	*hm66*	Nonsense (*lf*)	V: 11,415,735	R498stop
*des-2* + *deg-3*	*u773*	Deletion (*lf*)	V: 11,416,287–11,413,832	DES-2 is likely to be truncated after S492 and DEG-3 is eliminated
*deg-3*	*u662*	Missense(*gf*)	V:11,414,015	I314N
*deg-3*	*u701*	Deletion (*lf*)	V: 11,414,319–11,413,596	E231-G439
*deg-3*	*hm12*	Missense (*dn*)	V:11,414,042	G305E
*lgc-12*	*ok3546*	Deletion (*lf*)	III: 7,562,606–7,563,349	Frame shift after N119

⁎ Genetic effects of the mutations are given in parenthesis, loss of function (*lf*), gain of function (*gf*), or dominant negative (*dn*).

⁎⁎ Nucleotide positions are for the plus strand although *deg-3* and *des-2* are encoded by the minus strand.

⁎⁎⁎ Amino acid numbering is relative to the first methionine.

DEG-3 and DES-2 express at high levels in PVD and localize to all its processes, both dendrites (Fig. 1) and axon (not shown). Thus it is possible for these proteins to have a structural role in PVD. To examine this possibility we used a fourth mutation *hm12*, a missense mutation eliminating DEG-3 function without altering its distribution (Fig. 2A, Table 1, and Yassin et al., 2002). This mutation, like mutations affecting *des-2*, eliminates the behavioral response to harsh touch when combined with a mutation eliminating TRNs (Fig. 2B). Thus we suggest that PVD mechanosensation requires DES-2 and DEG-3 functioning as ion channel subunits and not as structural proteins. The smaller defect seen in the *lf*, *deg-3(u701)*, mutants relative to *deg-3(hm12)* mutants suggests a dominant negative (*dn*) effect of the *hm12* mutation, leading to sequestration of DES-2 into non-functional complexes.

MEC-10 and DEGT-1 were suggested to function as mechanosensors since they are needed for PVD's calcium response to high-threshold mechanical stimuli (Chatzigeorgiou et al., 2010). To better understand the roles of *deg-3* and *des-2* in PVD's mechanosensory response we similarly analyzed the calcium response of mutants defective for these genes to high-threshold mechanical stimuli. Results of this assay are similar to results of the behavioral assay, showing that both the *des-2deg-3(u773)* and *des-2(hm71)* mutations reduce PVD's calcium response to high threshold mechanical prodding, while the *deg-3(u701)* mutation leads to smaller non-significant reduction of this response (Fig. 2D). However, this assay, unlike the behavioral assay, shows reduction but not elimination of the response in *des-2deg-3(u773)* and *des-2(hm71)* mutants. Thus, DES-2, unlike MEC-10 and DEGT-1, is not essential for PVD's calcium response to mechanical stimuli. This difference is likely to be significant as the same calcium reporter and experimental setup used to analyze *mec-10(lf)* mutants were used to examine the nAChR subunit mutants (Chatzigeorgiou et al., 2010). Therefore, DES-2 appears to be required for sensitizing PVD or for amplifying PVD's response to mechanical stimuli, thus enabling a sufficiently large response needed to elicit PVD-mediated behavioral responses to harsh-touch.

To examine whether ACh is also required for enhanced responses to high-threshold mechanical stimuli we examined these responses in *cha-1(p1152)*; this mutation greatly reduces the activity of Choline Acetyltransferase, leading to reduced ACh synthesis and many defects in behavior and growth (Rand and Russel, 1984). Analysis of this mutant shows a significant reduction in PVD's calcium response to high threshold mechanical prodding (Fig. 2E) Therefore, supporting the suggestion that ACh-dependent activation of nAChRs enhances PVD's response to high-threshold mechanical stimuli.

### LGC-12 is a PVD expressed nAChR subunit functioning with DEG-3 and DES-2

**Fig. 3:**
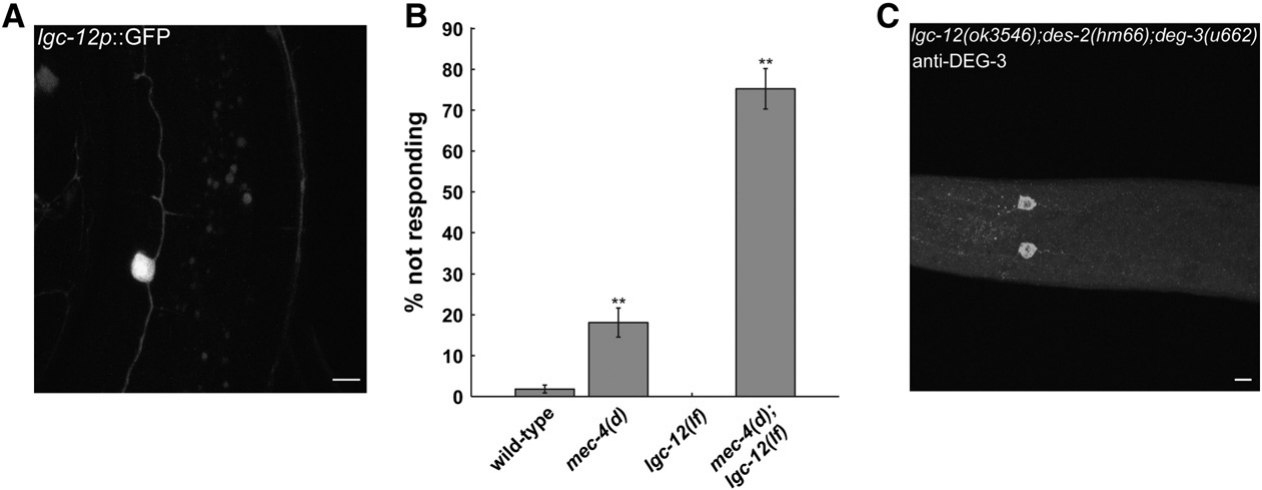
LGC-12 is expressed and functions in PVD. A) *lgc-12p*::GFP reporter is expressed in PVD as seen in an L4 animal. B) Harsh touch response of *mec-4 (d)*;*lgc-12(ok3546)* mutants is reduced relative to wild-type, *lgc-12(ok3546)*, or *mec-4(d)* mutants (N = 10–12,n = 10, p < 0.01, one-way ANOVA). C) Survival of PVD in *lgc-12(ok3546)des-2(hm66)deg-3(u662)* mutants in an adult animal. We note that trafficking of DEG-3 onto PVD processes is reduced in *des-2 (lf)* mutants (Yassin et al., 2002). Scale bar 5 μm.

Heterologous expression studies did not show functional channel formation when DES-2 was expressed alone (Treinin et al., 1998). However, results described above (Fig. 2) suggest that DES-2 is capable of forming a functional PVD-expressed channel in the absence of DEG-3. Similarly, neuronal degenerations resulting from a gain-of-function (*gf*) mutations in *deg-3*, *deg-3(u662)*, are reduced but not eliminated in *des-2 lf* mutants ((Treinin et al., 1998) and Table 1). Therefore, in *C. elegans* both DES-2 and DEG-3 are likely to combine with a yet unidentified nAChR subunit to form redundantly functioning complexes. A candidate for such a subunit is LGC-12, encoding for a nAChR subunit that does not belong to the 5 “core” groups of *C. elegans* nAChRs (Jones and Sattelle, 2003). LGC-12 appears high on the list of PVD enriched genes (11 fold enrichment (Smith et al., 2010)). To validate expression of *lgc-12* in PVD we examined expression of a *lgc-12* promoter fusion to GFP (*lgc-12p*::GFP) (Topalidou and Chalfie, 2011); this analysis shows expression of LGC-12 in PVD (Fig. 3A). We note that *lgc-12p*::GFP is not seen in PVD side branches probably due to its low expression in PVD. Thus analysis of a functional LGC-12::GFP fusion or antibody staining of LGC-12 is needed to examine its cellular localization. To examine whether LGC-12, like DEG-3 and DES-2, is required for the response to harsh touch we examined the harsh touch response of animals lacking *lgc-12* function (Table 1) or both TRNs and *lgc-12* function. This analysis (Fig. 3B) shows a significant reduction in the harsh touch response of the double mutant *mec-4(d)*;*lgc-12(ok3546)*. Thus, LGC-12, like DEG-3, is needed but is not essential for the PVD-mediated behavioral response to harsh touch.

To examine the role of LGC-12 in *deg-3(u662)*-dependent degenerations we examined the number of swollen neurons in early L1 larvae (a hallmark for *deg-3(u662)*-dependent degenerations (Treinin and Chalfie, 1995)). For this analysis we examined the effects of the *lgc-12*/(*lf*) mutation on degenerations seen following partial suppression of *deg-3(u662)*-dependent degenerations by the *des-2(hm66)*, a DES-2 *lf* mutation (Table 1). This analysis shows significant reduction in the number of swollen cells in *lgc-12(ok3546)des-2(hm66)deg-3(u662)* relative to *des-2 (hm66)deg-3(u662)* (0.08 relative to 0.5 swollen cells per animal, n = 46, 22 respectively, p < 0.01). Importantly, degeneration of PVD occurring later in development is highly efficient in *des-2(hm66)deg-3(u662)* animals (no surviving DEG-3 stained PVDs are seen in adult animals) while in *lgc-12(ok3546)des-2(hm66)deg-3(u662)* surviving, DEG-3 stained, PVD neurons are seen (Fig. 3C).

Together, our results suggest that in PVD LGC-12 interacts with DES-2 to form a receptor needed for the harsh touch response or with DEG-3*(u662)* to form a degeneration causing receptor. Moreover, our results suggest that PVD expresses several different nAChR compositions: DEG-3/DES-2, DEG-3/LGC-12, and DES-2/LGC-12; receptor compositions likely to have different properties and functions. In addition, LGC-12 when expressed alone is sufficient for formation of an ACh-gated channel in *Xenopus* oocytes (Saras, 2005). Thus PVD may also express a homomeric LGC-12 receptor.

### DEG-3 and DES-2 are required for the response to cold temperatures

**Fig. 4:**
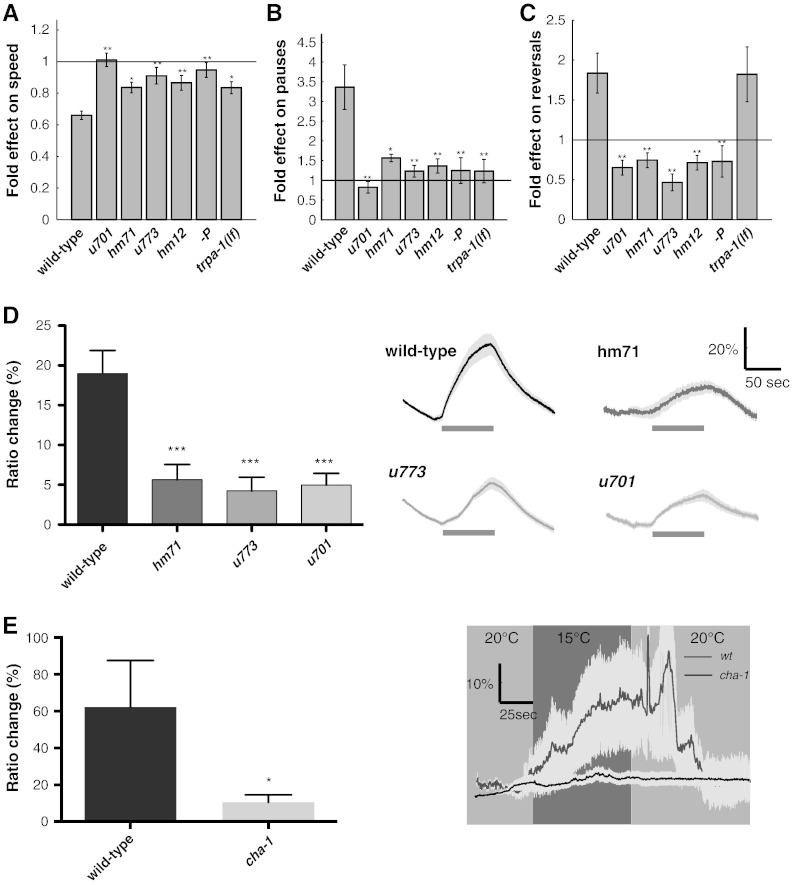
The response to cold temperature requires DEG-3 and DES-2. A–C) Mutants were examined for locomotion properties at 20 °C or 15 °C. Shown is the ratio between locomotion properties measured in 15 °C relative to 20 °C. A) Speed. B) Percent time in pause. C) Rate of reversals. Number of animals is 36, 20, 18, 19, 20, 17, 15 in order of appearance. D) Left, averaged peak calcium response in wild-type, *des-2(hm71)*, *des-2 deg-3(u773)*, and *deg-3(u701)*, N = 16, 13, 25, 15 respectively. Differences between all mutants and wild-type are significant p < 0.001 one-way ANOVA. Right, averaged traces of calcium responses with s.e.m. Bars indicate time of temperature downshift. E) Left, averaged peak calcium response in wild-type and *cha-1(p1152)*, N = 6, 14 respectively. Right, averaged traces of calcium responses with s.e.m. bars indicate time of temperature downshift. Significant difference relative to wild-type were examined using one-way ANOVA (* — p < 0.05, ** — p < 0.01).

Previous work showed altered locomotion of PVD-ablated animals relative to wild-type animals at 20 °C (Albeg et al., 2011). Similar analysis of animals transferred to 15 °C shows many temperature-dependent changes in locomotion, changes that depend on PVD (Fig. 4A–C). Indeed, PVD was previously shown to mediate the response to a sharp decrease in temperature in a TRPA-1-dependent manner (Chatzigeorgiou et al., 2010). This previous study examined the immediate response to a sharp and transient temperature change while our assay examines locomotion 10 min following transfer to a new temperature; therefore, the effects of temperature seen in this assay may represent an enduring response to reduced temperature. To examine whether locomotion properties measured in both assays depend on the same molecular mechanisms we examined locomotion properties of *trpa-1(lf)* at 20 °C and 15 °C. Results shown in Fig. 4A,B show that, in this assay, effects of temperature on speed and on the fraction of time pausing require TRPA-1. To examine whether effects of temperature on locomotion, like the effects of harsh-touch, require DEG-3 and DES-2 we looked at effects of temperature on locomotion of *deg-3* and *des-2* mutants. Analysis of speed, reversal rates, and fraction of time spent in pauses show that both *deg-3* and *des-2* are required for these temperature-dependent changes in locomotion properties (Fig. 4A–C). Thus DEG-3 and DES-2 function in PVD-mediated responses to mechanical and thermal stimuli.

Interestingly, while effects of temperature on reversal rates require PVD, DEG-3, and DES-2 they do not require TRPA-1 (Fig. 4C). TRPA-1 was shown to mediate PVD's calcium response to cold and the PVD-mediated behavioral response to cold, both of which were measured immediately following temperature reduction (Chatzigeorgiou et al., 2010). Thus the molecular mechanisms underlying the immediate and the late responses to cold are similar but not identical. To examine whether DEG-3 and DES-2 are also required for the immediate response to cold we used calcium imaging as previously described (Chatzigeorgiou et al., 2010). Results in Fig. 4D show that the calcium response to cold is greatly reduced in *deg-3* and *des-2* mutants. And, as was shown for the response to mechanical stimulation, *deg-3* and *des-2* mutations do not eliminate this response. Similarly, mutation in *cha-1* (Rand and Russel, 1984), needed for synthesis of ACh, also reduces PVD's response to cooling (Fig. 4E). The difference between calcium responses seen in nAChR subunit and ACh synthesis mutants relative to the *trpA-1* mutant is likely to be significant as the same calcium reporter and experimental setup were used for analyzing the response to cooling in all these mutants (Chatzigeorgiou et al., 2010). Thus, ACh and nAChR subunits may function to sensitize or amplify the TRPA-1-mediated response to cold. Moreover, unlike TRPA-1, DEG-3 and DES-2 are also needed for the late PVD-mediated effect on reversals (Fig. 4C). This suggests that DEG-3 and DES-2 may also enhance responses of an additional cold sensor in PVD, a sensor whose effects on behavior are only seen following longer exposure to cold. Last, unlike the responses to mechanical stimuli, responses to cold do not differ between the different *deg-3* and *des-2* mutations. Therefore, nAChRs containing different subunit combinations are differentially required for different sensory modalities.

### PVD morphology defects in animals lacking nAChR subunits

**Fig. 5:**
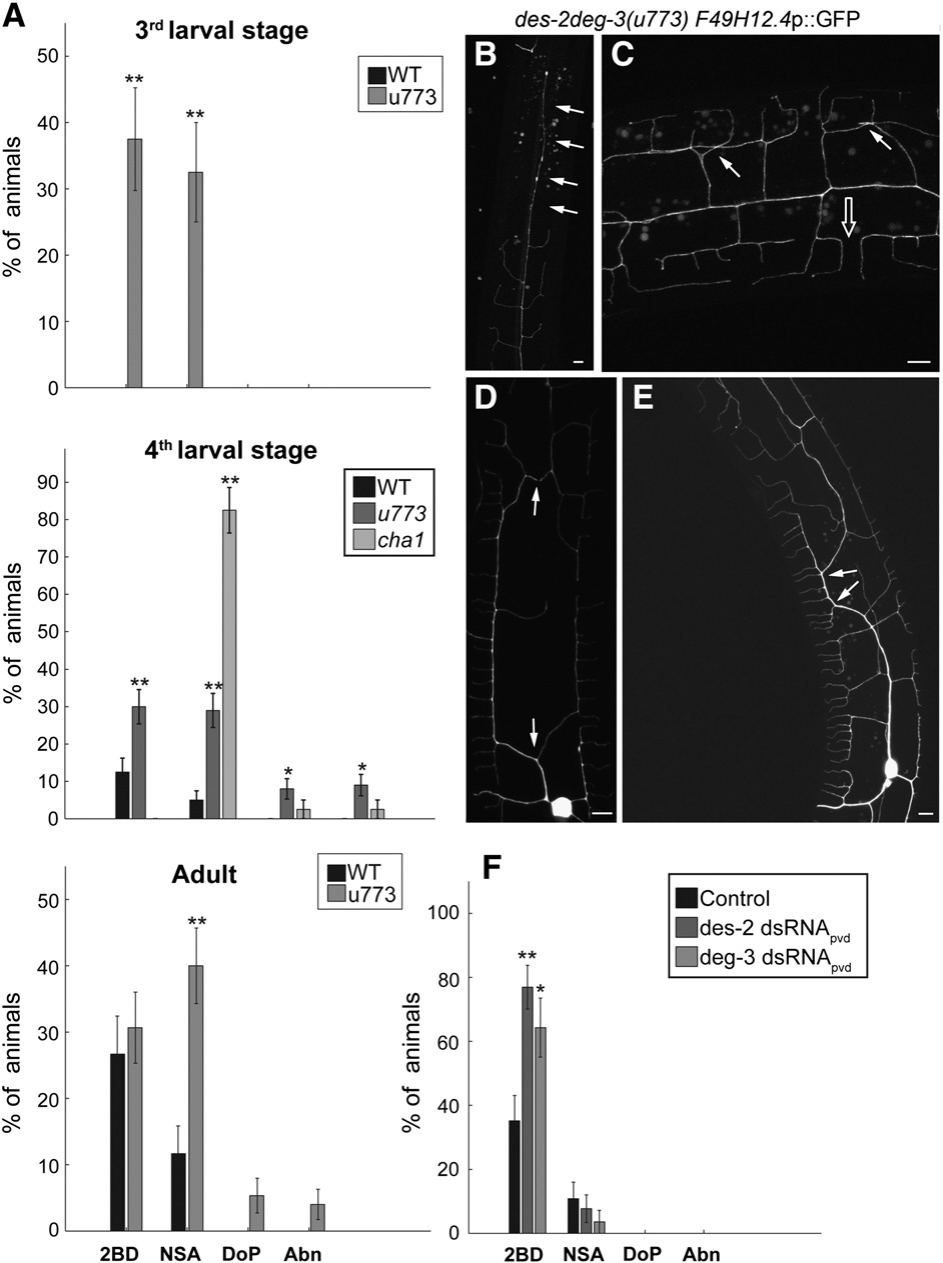
nAChR subunits are required for normal PVD morphology. A) Defects in PVD morphology of *deg-3des-2 (u773)* and *cha-1(p1152)* at different stages of development (due to the very slow growth of *cha-1* mutants they were only examined at L4). Analysis of percent animals having defects in: the distribution of secondary branches (less secondary branches emerging from distal ends of primary, 2BD), defects in self avoidance (NSA), discontinuities of the primary branch (DoP), grossly abnormal morphology (Abn). Animals examined at L3 = 20, 40; L4 = 80, 100, 40; Adult = 60, 75; wild-type, *des-2deg-3(u773)*, and *cha-1(p1152)* respectively. Significant differences relative to wild-type are indicated by * — p < 0.05, ** — P < 0.01, *t*-test. B–E) Example of PVD morphology defects in *des-2deg-3(u773)* mutants. B) Reduced number of secondary branches emerging from the distal ends of the primary branch, position of missing branches indicated by arrows. C) Self avoidance defects. Arrows indicate overlapping tertiary branches and empty arrow indicates an example of normal self-avoidance. D and E) Examples of grossly abnormal morphologies of primary branches. Abnormalities indicated by arrows. all images are of late L4-young adults, scale bar 5 μm. F) Cell autonomous effects of nAChR subunits on secondary branch formation. Numbers are given for L4-young adults of *ser-2prom3* (empty vector, controls), *ser-2prom3*:*des-2* dsRNA, and *ser-2pro3*:*deg-3* dsRNA, n = 37, 39, 28 respectively. Significant differences relative to controls are indicated by * — p < 0.05 ** — p < 0.01, one-way ANOVA.

Mutants lacking *deg-3* and *des-2* are defective for both known PVD sensory modalities (mechanical and thermal). Such defects may be secondary to defective development of PVD's arbor. To examine this possibility we expressed *F49H19.1p*::GFP reporter in *des-2deg-3(u773)* mutants. This reporter is highly expressed in PVD and therefore enables detailed analysis of PVD development and morphology (Smith et al., 2010). The majority of *des-2deg-3(u773)* animals examined using this reporter show no gross abnormalities in PVD morphology. Thus defective PVD morphology is an unlikely cause for the functional defects seen in this mutant. However, detailed analysis of this strain does identify the following defects: Secondary branches appear during the third larval stage (L3) growing from the primary branch in a proximal to distal order (relative to the PVD cell body) (Smith et al., 2010). In many *des-2deg-3(u773)* mutant animals (40% of L3 animals n = 40), the anterior distal ends of the primary branch lack secondary branches (Fig. 5A, B). This defect is not seen in wild-type controls from the same larval stage. Importantly, this difference is seen in the third and fourth larval stages (L3 and L4) of animals and disappears in adults, suggesting that it represents delayed development of PVD. The second defect observed is in tertiary branches. These branches appear late in L3. Starting to grow at L3 and continuing in L4. Tertiary branches from adjacent menorahs grow towards each other but their overlap is avoided via active retraction (Smith et al., 2010, Smith et al., 2012). In *des-2deg-3(u773)* mutants overlapping tertiary branches are seen, starting in L3 and persisting into adulthood (Fig. 5A,C). The early appearance of this defect suggests a developmental defect in tertiary branch retraction enabling self-avoidance (Smith et al., 2012).

In a small fraction of *des-2deg-3(u773)* animals, we observe grossly abnormal PVD arbors (Fig. 5A,D,E). These defects are seen late in development (L4 and young adults) and are thus unlikely to represent defects in development and may instead represent defects in arbor maintenance (Fig. 5A). Interestingly, in mutant L4 and young adult animals, but not earlier in development, we also observe low frequency discontinuities in PVD primary branches that may represent breaks in these processes (Fig. 5A). This defect may also represent defective maintenance of PVD dendrites.

To examine whether different nAChR subunits have distinct or partly overlapping roles in PVD development we examined PVD morphology in *deg-3(u701)* and *des-2(hm71)* single mutants, and in *des-2deg-3(u773)*;*lgc-12(ok3546)* double mutants. This analysis showed similar defects in the different mutants (Supplemental Fig. 1). To examine whether *deg-3* and *des-2* are required cell autonomously we also analyzed strains expressing *des-2* or *deg-3* dsRNA in PVD. These strains show defects in distribution of PVD secondary branches similar to those seen in *des-2deg-3(u773)* animals (Fig. 5F). Together, our results suggest that DEG-3 and DES-2 function together in PVD to affect development of PVD branches.

Last we examined the effects of reducing ACh synthesis on PVD morphology. This analysis (Fig. 5A and Supplemental Fig. 2) shows similar and even higher fraction of animals showing defects in self-avoidance of tertiary branches. Therefore, supporting the suggestion that self-avoidance requires ACh-dependent activation of nAChRs. However, other aspects of morphology differ between the *cha-1* and nAChR subunit mutants (Fig. 5A and Supplemental Fig. 2). Such differences can be attributed to the complexity of cholinergic signaling mechanisms.

### DEG-3 and DES-2 are needed for amplifying optogenetic stimuli

**Fig. 6:**
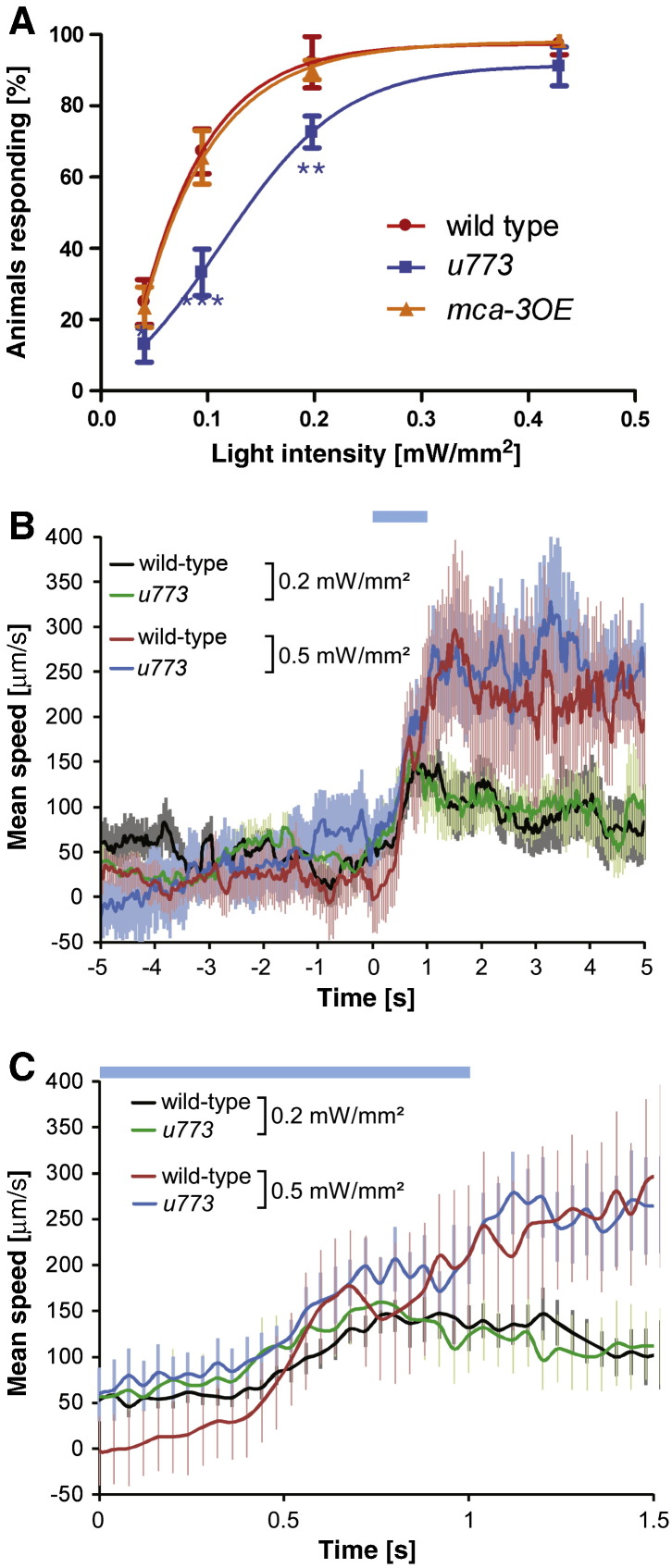
nAChR subunits affect transmission of light induced signals in PVD. A) Percent of animals, wild-type, *des-2deg-3(u773)*, and MCA-3 overexpressing, responding to brief light stimuli at different light intensities, N = 4–5 trials, n = 26–40 animals/trial. Significant differences between *u773* and wild-type are indicated as * — p < 0.05, ** — P < 0.01, *** — P < 0.001, *t*-test. B) Speed of reacting animals in response to brief light stimuli. Only responding animals are analyzed for wild-type or *des-2deg-3(u773)* at 0.5 mW/mm^2^ and 0.2 mW/mm^2^ (n = 10, n ≥ 8 for all data points). C) Magnification of the first 1.5 s from B, Time = 0 s indicates start of a 1 s blue light stimulus, which is represented by the blue bar.

Results described above show that nAChRs are needed for several distinct processes occurring in PVD neurons. Two of these processes were suggested to depend on DEG/ENaC and TRP channels; mutations in the DEG/ENaC, *mec-10*, or in *trpa-1* abolish calcium responses to mechanical or thermal stimuli, respectively (Chatzigeorgiou et al., 2010). These effects differ from effects of mutations in nAChR subunits that reduce but do not abolish calcium responses to sensory stimuli (Fig. 2, Fig. 4). Therefore, nAChRs may function to amplify responses to sensory stimuli detected by PVD. To examine this possibility we examined the behavioral response to light induced activation of channelrhodopsin (ChR2) expressed in PVD. Such analysis has previously identified proteins functioning downstream of nociceptive sensors; mutations or dsRNA-mediated knockdown of these genes reduced or attenuated the behavioral response to optogenetic activation of PVD (Husson et al., 2012). Analysis of *des-2deg-3(u773)* animals expressing ChR2 in PVD shows a reduction in the behavioral response to light stimuli, which is dependent on the light intensity used (Fig. 6A). Specifically, no or a small (20%) decrease in percentage of animals responding is seen at the higher light intensities and a larger (approximately 50%) decrease is seen at the two lower light intensities. Detailed analysis of speed in responding animals shows no difference in maximal speed or in the kinetics of the response to light stimuli (Fig. 6B and Supplemental Fig. 3). Therefore, nAChRs may be required downstream of nocisensors to reliably transmit weak signals.

### Reduced calcium levels in nAChR mutants may explain PVD defects

**Fig. 7:**
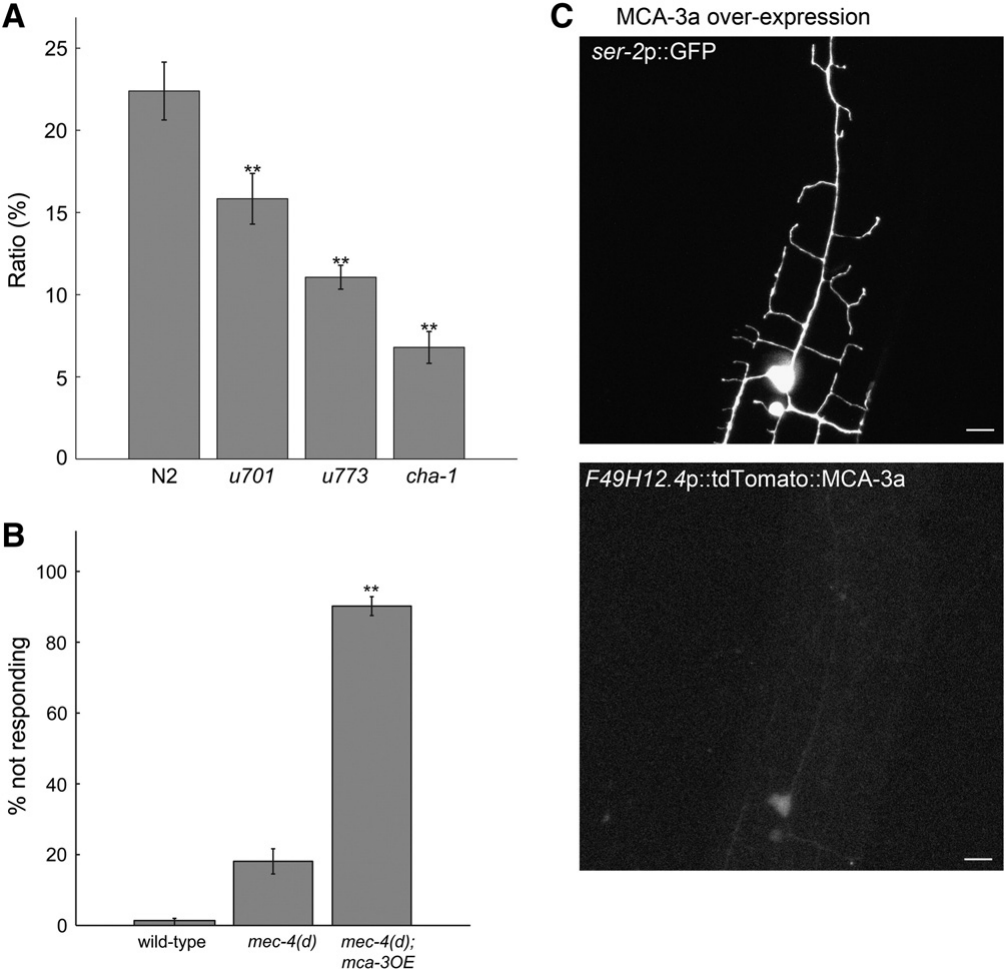
Reduced cytosolic calcium in nAChR mutants may explain PVD defects. A) Basal calcium levels in wild-type, *deg-3(u701)*, *des-2deg-3(u773)* and *cha-1(p1152)* animals n = 117, 76, 108, 27 respectively. Differences between mutants and wild-type are significant p < 0.01, one-way ANOVA. B) MCA-3 overexpression leads to a harsh touch defect. Differences between the *mec-4(d)*; MCA-3 overexpressing strain and either *mec-4(d)* or wild-type (N2) are significant, N = 10–12, n = 10, p < 0.01, one-way ANOVA. C) Above MCA-3 overexpression leads to defects in PVD morphology. Below, distribution of the functional tdtomato::MCA-3a protein in the same cell. Shown are images of late L4-young adults, scale bar 5 μm.

Results described above show that ACh and nAChRs are needed for both function and morphology of PVD. The DEG-3/DES-2 nAChR was shown to have high calcium permeability (Yassin et al., 2001); Calcium permeating via DEG-3 and DES-2 containing channels has the potential to affect multiple processes and proteins and therefore may explain the defects caused by reduced nAChR-dependent cholinergic signaling. To examine whether mutations in DEG-3 and/or DES-2 are associated with reduced cytosolic calcium we estimated calcium levels of wild-type and mutants in the absence of acute external stimulus (basal calcium). Such analysis (Fig. 7A) shows reduced basal calcium levels in both *deg-3(u701)* and *des-2deg-3(u773)* mutants relative to wild-type. Mutation in *cha-1*, needed for ACh synthesis, leads to an even larger reduction in basal calcium (Fig. 7A). Thus ACh-dependent activation of nAChRs increases basal calcium level in PVD. The lower basal calcium level seen in *cha-1(p1152)* mutants relative to the nAChR subunit mutants, suggests that additional ACh-dependent signaling pathways may function in PVD to maintain basal calcium levels.

We hypothesize that reduced cytosolic calcium in nAChR mutants is responsible for the defects seen in these mutants. To examine this hypothesis we analyzed animals having reduced cytosolic calcium due to PVD overexpression of a calcium pump, MCA-3 (Bednarek et al., 2007). Results of this analysis show that MCA-3 overexpression leads to: 1. Reduced response to harsh touch when combined with the *mec-4(d)* mutation (Fig. 7B). 2. Large defects in PVD morphology (Fig. 7C). 3. No reduction in the behavioral response to optogenetic stimulation of PVD (Fig. 6A). Thus a reduction in PVD calcium levels mimics the effects, on a PVD-mediated behavioral response and on PVD morphology, of reducing nAChR activity. Moreover, the reduced behavioral response to harsh touch cannot be attributed to processes occurring downstream to mechanosensors' activity, such as reduced neurotransmitter release. However, we note that MCA-3 overexpression does not precisely mimic the effects of nAChR mutations. Specifically, effects of MCA-3 overexpression on secondary branch development are more severe when compared to nAChR mutants. In addition, while deletion of *deg-3* and *des-2* interferes with processes occurring downstream of the nocicensors, MCA-3 overexpression has no such effect (Fig. 6A). We attribute these differences to compartment specific differences in calcium levels in the different strains. These differences may be a result of different distribution of the proteins: DEG-3 and DES-2 are found all over PVD (cell body, proximal and distal branches) while distribution of the tagged MCA-3 protein is not seen in distal branches (Fig. 7C). Moreover, within the cell body where MCA-3 is expressed its effects on calcium levels are likely to be stronger than effects of eliminating nAChRs, as these are likely to represent only one venue for calcium entry.

## Discussion

Polymodal nociceptors sense high threshold thermal or mechanical stimuli. Most polymodal nociceptors also respond to chemical signals. These chemicals are diverse and elicit diverse responses including sensitization to other stimuli (Fischer et al., 2010). PVD is a *C. elegans* nociceptor shown to respond to high-threshold mechanical stimuli and to cold temperatures (Chatzigeorgiou et al., 2010). Work presented here shows that ACh and subunits of ACh-gated ion channels are required for effective PVD responses to mechanical or thermal stimuli. We also demonstrate a role of nAChRs in the development and maintenance of PVD's dendritic arbor. These defects in PVD morphology are minor and unlikely to explain sensory defects. Taken together, our results are consistent with ACh activating nAChRs to increase cytosolic calcium being required for effective signaling within PVD. However, we cannot rule out calcium independent mechanisms of action of ACh and nAChRs.

The nAChRs are a large, diverse and evolutionarily conserved family of neurotransmitter-gated channels, best known for the muscle-expressed nAChR that is essential for muscle excitation. However, nAChR function is not limited to synaptic excitation; in fact, members of this family are expressed widely in the central and peripheral nervous system as well as outside the nervous system and control multiple processes including, synaptic vesicle release, neuroprotection, and development (Kalamida et al., 2007, Kurzen et al., 2007). These non-synaptic effects are likely dependant on calcium permeating through nAChRs and regulating various signaling pathways (Dajas-Bailador and Wonnacott, 2004). Evidences for the role of calcium permeating via DEG-3- and/or DES-2-containing channels in PVD function and morphology are as follows: 1. In the absence of acute stimuli, cytosolic calcium levels are lower in nAChR subunit and ACh synthesis mutants than in wild-type. 2. Overexpression of a calcium pump in PVD mimics phenotypes seen in nAChR subunit mutants. Thus we suggest that calcium permeating via DEG-3- and/or DES-2-containing channels affects PVD signaling pathways. We note that increased cytosolic calcium levels are associated with, and are in fact required for, sensitization of mammalian nociceptors (Guenther et al., 1999). Thus, signaling pathways regulating cytosolic calcium levels are likely to constitute a conserved mechanism regulating the response to noxious stimuli.

Mammals, like *C. elegans*, express nAChRs in sensory neurons. A well-known example is the α9α10 nAChR expressed in hair cells where it has a role in auditory processing (Elgoyhen et al., 1994, Elgoyhen et al., 2001). Other nAChRs are expressed in dorsal root ganglion (DRG) neurons, including nociceptors (Genzen et al., 2001, Hone et al., 2012, Rau et al., 2004, Spies et al., 2006). Evidence for the role of nAChRs in nociception comes from nAChR agonists and antagonists being developed as treatment for pathological pain. However, the site of action of these drugs is not always clear and is likely to include targets in the central nervous system, peripheral branches of nociceptors, and cells belonging to the immune system (Damaj and Flores, 2001, Vincler et al., 2006). In addition, ACh applied to the periphery elicits responses in nociceptors (Armstrong et al., 1953). However, analysis of this response suggests involvement of both nicotinic and muscarinic ACh receptors (Bernardini et al., 2001). Specifically, nicotinic receptors mediate the sensitizing effects and muscarinic receptors mediate the stronger and longer lasting desensitizing effects of ACh on these neurons (Bernardini et al., 2001). Likewise, complexity of cholinergic signaling in PVD may explain differences in phenotypes of mutants and transgenics used in this study; indeed, we show that PVD expresses three nAChR subunits likely to form different nAChRs having different subunit compositions, properties, and functions; in addition, PVD was shown to express muscarinic acetylcholine receptors (Smith et al., 2010).

Our results show that in *C. elegans* “normal” PVD morphology and PVD-mediated behavioral responses require nAChR activity. This suggests constant exposure of PVD sensory dendrites to ACh. One source of ACh, needed to activate PVD expressed nAChRs, is spillover from the neuromuscular junctions (NMJs), residing in the ventral and dorsal cords adjacent to the terminal endings of PVD dendrites (Albeg et al., 2011). A second source is the hypodermis residing just above PVD terminal dendrites (Albeg et al., 2011); the hypodermis of *C. elegans* expresses choline acetyltransferase (ChAT (CHA-1), the enzyme responsible for ACh synthesis) (Duerr et al., 2008) and the hypodermis of *Ascaris*, a closely related nematode, shows low-level ChAT activity (Johnson and Stretton, 1985). In mammals sources of ACh needed to activate nAChRs expressed on peripheral dendrites of nociceptors are keratinocytes, DRG neurons themselves, and immune cells (Kurzen et al., 2007, Sann et al., 1995, Wessler et al., 1999).

Our results, showing reduced noxious stimuli induced calcium influx in mutants defective for ACh synthesis or nAChR subunits, suggest that nAChR activity facilitates signaling within PVD. We also show that nAChRs increase the response to weak optogenetic stimuli. This last result is consistent with enhanced signaling downstream of nocisensors. However, the strong behavioral defects in the response to noxious stimuli seen in nAChR mutants suggest additional effects, such as direct effects on responsiveness of the DEG/ENaC mechanosensors and TRPA-1 cold sensors (Chatzigeorgiou et al., 2010, Smith et al., 2012).

In mammal chemical signals, released following inflammation or injury were shown to sensitize mammalian nociceptors (Fischer et al., 2010, Gold and Gebhart, 2010). And ACh functioning via nAChRs was shown to sensitize mammalian nociceptors (Bernardini et al., 2001). Whether injury increases levels of ACh to activate DEG-3- and/or DES-2-containing channels and to further sensitize PVD, is yet unknown. One possibility is that injury leads to the release of ACh from damaged hypodermal cells. A similar mechanism, i.e. ACh release from damaged keratinocytes, may explain the presence of ACh among “inflammatory mediators” of mammals (Grando et al., 1993). In addition, noxious stimuli were found to regulate ACh release from keratinocytes (Nagy et al., 2006). Overall our findings concerning the roles of ACh and nAChRs in *C. elegans* PVD neurons together with the many studies on ACh and nAChRs in mammalian nociceptors suggest a conserved role for nAChR mediated cholinergic transmission in controlling responsiveness of polymodal nociceptors.

## Experimental methods

### Strains and mutations

The wild-type is N2. Most mutations and transgenics have been previously described: In brief: in *mec-4(d)* mutants, the *e1611* allele, TRNs degenerate (Driscoll and Chalfie, 1991); in *ser-2prom3*:DEG-3-N293I expressing animals PVD degenerate (-P animals) and in *mec-10p*::DEG-3-N293I animal PVD, FLP, and TRNs degenerate (-TPF animals)(Albeg et al., 2011); the *trpA-1(lf)* strain is RB1052. The *u662* mutation was originally named N293I (293 is the position of the mutation in the mature protein, after cleavage of the signal peptide), however current nomenclature is I314N ((Treinin and Chalfie, 1995) and Table 1.). The *hm66* mutation was identified as a suppressor of *deg-3(u662)*-dependent degenerations (Treinin et al., 1998). Sequence of the *hm66* and *hm71* mutations was obtained following PCR amplification of *des-2* from mutant animals. Molecular analysis of other *deg-3* and *des-2* was previously described (Treinin and Chalfie, 1995, Yassin et al., 2002). In Table 1 and throughout the text mutations were assigned into types (*lf*, *gf*, or *dn*) according to genetic and molecular analyses. In addition, anti-DEG-3 staining (the antibody recognizes the last amino acids of DEG-3) of *deg-3(u701)* and *des-2deg-3(u773)* animals detected no protein, confirming their status as DEG-3 loss of function mutations. For calcium imaging studies we used the previously described calcium reporter transgene (ljEx19[p*egl-46*::YC2.3 lin-15 (+)]) (Albeg et al., 2011, Chatzigeorgiou et al., 2010) and for optogenetics we used the inserted transgene, zxIs12 (p*F49H12.4*::ChR2::mCherry;p*F49H12.4*::GFP) (Husson et al., 2012).

### Immunohistochemistry and molecular biology

DEG-3 antibodies and staining were previously described (Yassin et al., 2001). The full-length DES-2::GFP fusion is described in (Oren-Suissa et al., 2010), the truncated DES-2::GFP fusion contains a 5-Kb XbaI-BamHI genomic fragment encoding for the *des-2deg-3* promoter followed by genomic *des-2* coding sequence and terminating in a BamHI restriction site residing in the middle of the large intracellular loop of DES-2 (Treinin et al., 1998). To knockdown expression of *des-2* or *deg-3* in PVD we used the method developed by Esposito et al. (2007) for expressing double stranded RNA of genes of interest in specific cells (Esposito et al., 2007). Specifically, coding inserts were amplified from the *C. elegans* RNAi library using primers containing a *Hind*III site (Source BioScience, clone V-7D14 (*des-2*) and V-7B18 (*deg-3*) (Kamath et al., 2003)) followed by cloning of 815 bp (*des-2*) or 1.1 kb (*deg-3*) *Hind*III fragments in both orientations downstream to a 1.7 Kb *ser-2prom3* fragment in pBluescript SKII(−); this promoter expresses in PVD and OLL (Tsalik et al., 2003). The resulting plasmids were injected at 50 ng/μl of each orientation together with a *ser-2prom3*::GFP reporter into N2 animals (Tsalik et al., 2003). For *mca-3* overexpression we inserted a 4266 bp MCA-3 ORF + *unc-54* UTR *Nhe*I–*Spe*I fragment from Pcc::GFP-MCA3a (Bednarek et al., 2007) downstream and in frame to a *Kpn*I–*Nhe*I tdtomato encoding fragment from pMT:tdTomato and a 2040 bp fragment encoding for the F49H12.4 promoter (from pCJS01). This construct was injected at 50 ng/μl with a *ser-2prom3*GFP marker. The *ser-2prom3*::*GFP* plasmid used as an injection marker also enabling visualization of PVD branches is a 1659 bp *Sph*I–*BamH*I *ser-2prom3* fragment inserted into pPD95.75 (Fire et al., 1990).

### Behavioral assays and optogenetics

For harsh touch analysis young adults were examined for their response to prodding the mid-body with a platinum wire. Animals reversing or increasing their forward speed were considered as responding (Way and Chalfie, 1989). For analysis of the effects of temperature on locomotion properties we transferred animals grown at 20 °C to plates pre-incubated at 20 °C or 15 °C. Animals were allowed to recover from the transfer and to acclimate to the new temperature for 10 min before their locomotion was analyzed as described in (Albeg et al., 2011). For optogenetics the *des-2deg-3(u773)* mutation was crossed with a line containing the integrated transgene zxIs12 (expressing pF49H12.1::ChR2::mCherry) and responses to blue light were examined as in (Husson et al., 2012). To quantify the fraction of reacting animals, we challenged ± 30 worms with a short blue light pulse (460–500 nm) and assayed the reactivity as an escape response. Each trial was repeated at least 4 times, using 4 different light intensities (0.429 mW/mm^2^, 0.198 mW/mm^2^, 0.095 mW/mm^2^ and 0.041 mW/mm^2^). For detailed analysis of the response to light stimuli worms were tracked using computer-controlled motorized xy-stage and light-evoked behavioral output was quantified using custom-written software (Stirman et al., 2011).

### Calcium imaging

To image calcium transients in PVD we used the previously described *egl-46p*::YC2.3 reporter (Chatzigeorgiou et al., 2010, Albeg et al., 2011) which was crossed into the different *deg-3*, *des-2*, or *cha-1* mutants. Responses to harsh touch or to cold were examined as in (Chatzigeorgiou et al., 2010). Basal calcium levels are estimated from the YFP/CFP ratio averaged over a 10 second interval in the absence of acute stimuli.

### Microscopy and staging

Synchronization: All adult and larval worms were washed off the plates with M9 solution. After 2 h newly hatched larvae were collected and transferred to new plates. Mounting: A 5 μL drop of M9 with 20 mM levamisole and 1% tricaine methanesulfonate was placed on an agar pad (2% agar). Worms were transferred to this drop; after paralysis, a cover slip was placed and sealed with 50%/50% paraplast/paraffine.

dsRNA-expressing animals and *cha-1* mutants were synchronized by eye as L4 or young adults. Images were taken using a Zeiss LSM710 confocal microscope.

### Statistical analysis

For analysis we used MATLAB and Statistics Toolbox Release 2012b, The MathWorks, Inc., Natick, Massachusetts, United States, 2012. When more than two variables were compared, the significance was tested using one way ANOVA and a multiple comparison test with a Bonferroni adjustment. When only two variables were compared, the significance was tested using a two sample *t*-test or Wilcoxon test. Outliers were found using the Outlier calculator from GraphPad software. N — is the number of experiments and n — the number of animals in each experiment.
