# Supplementary material for: Caenorhabditis elegans nicotinic acetylcholine receptors are required for nociception
Source: Mol Cell Neurosci. 2014 Mar;59:85–96. doi: 10.1016/j.mcn.2014.02.001 (PMC4258610; doi:10.1016/j.mcn.2014.02.001)
Supplement: Supplementary file 1 — Supplementary figures. [file mmc1.pdf]

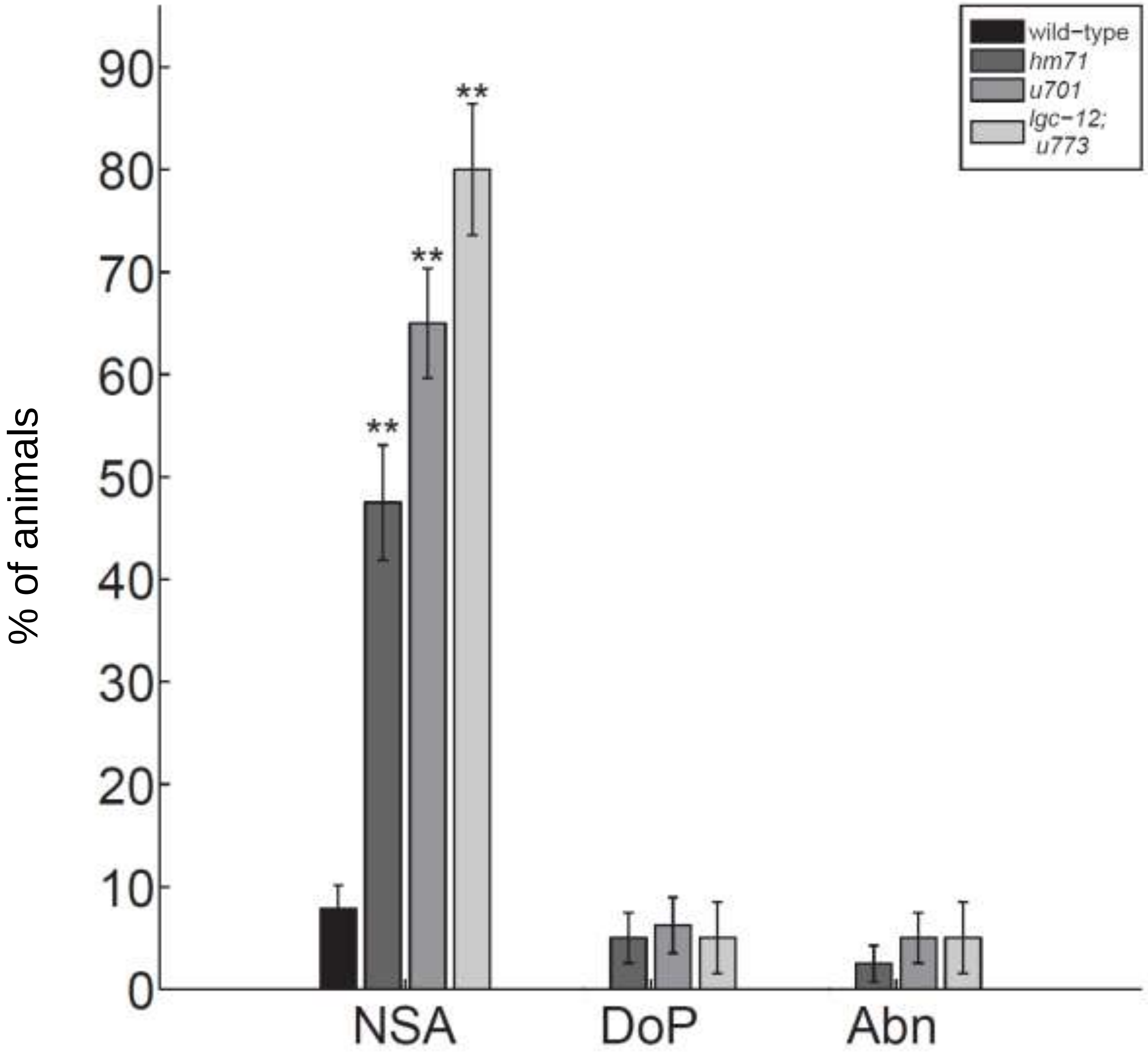

Supplemental Figure 1. Morphology of nAChR Mutants. Analysis of percent animals having: defects in self-avoidance (NSA), discontinuities of the primary branch (DoP), grossly abnormal morphology (Abn). Animals examined at the L4-adult stage. Wild-type (N=140), *deg-3(u701)* (N=80), *des-2(hm71)* (N=80), *des-2deg-3(u773)lgc-12(ok3546)* (n=40). Significant differences relative to wild-type are indicated by **- P<0.01, t-test.

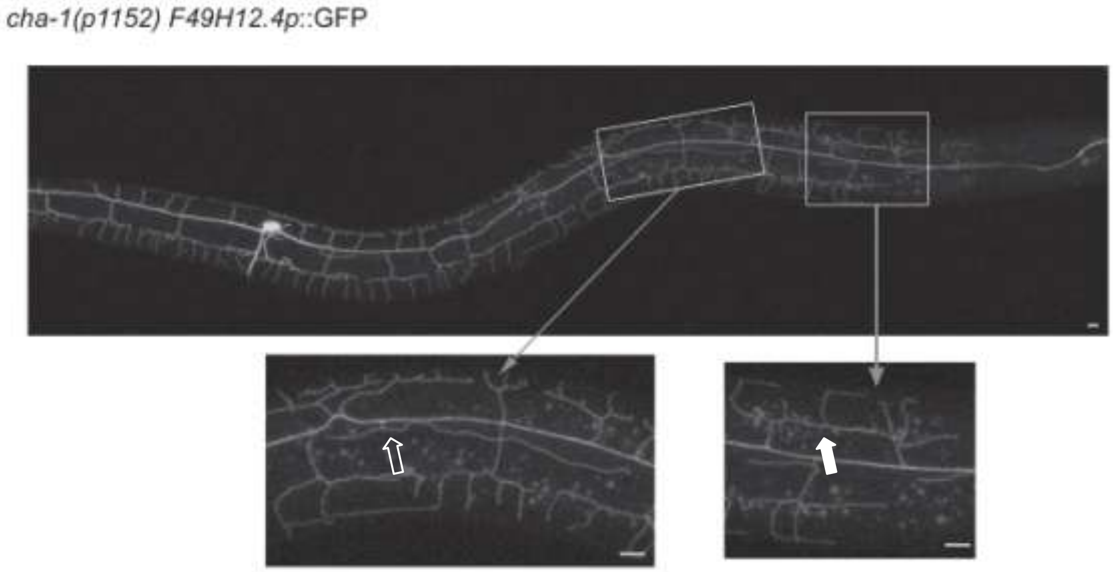

Supplemental Figure 2. Representative image of a *cha-1(p1152)* L4 animal. Filled arrows point to non-self avoidance; empty arrows indicate aberrant secondary branches. Scale bar 5µm.

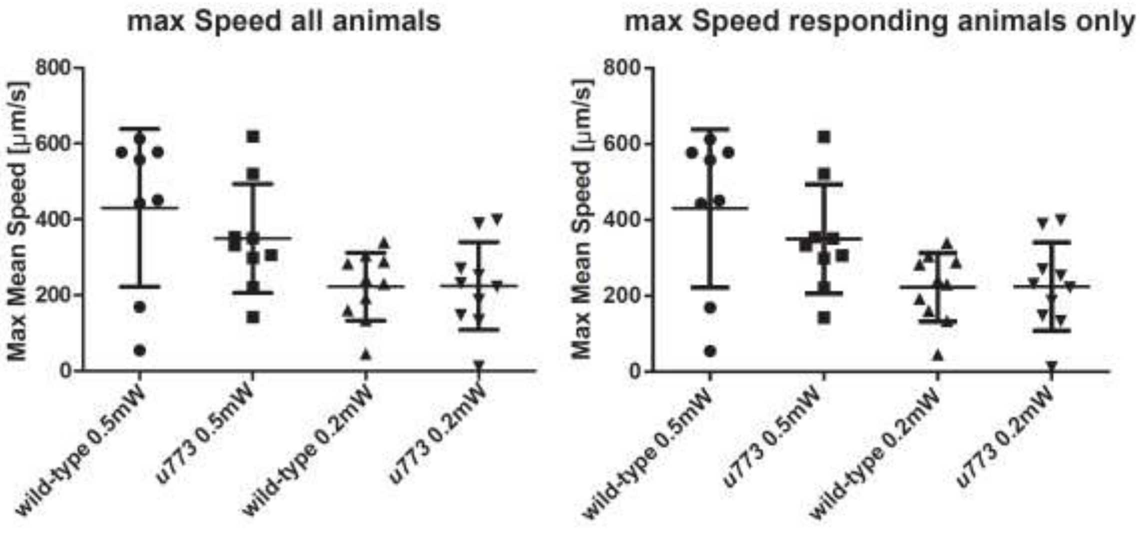

Supplemental Figure 3. Scatter graphs for maximal speed following illumination of wild-type (N2) and *des-2deg-3(u773)* mutants. Left all animals analyzed, right responding animals only.
